# Supplementary material for: Transcriptional profiling of extraocular motor neurons reveals sim1a as a candidate strabismus-related gene
Source: bioRxiv. 2026 Apr 8:2026.04.07.717009. Preprint. [Version 1] doi: 10.64898/2026.04.07.717009 (PMC13081939; doi:10.64898/2026.04.07.717009)
Supplement: Supplement 1 [file NIHPP2026.04.07.717009v1-supplement-1.pdf]

bioRxiv preprint doi: https://doi.org/10.64898/2026.04.07.717009; this version posted April 8, 2026. The copyright holder for this preprint (which was not certified by peer review) is the author/funder, who has granted bioRxiv a license to display the preprint in perpetuity. It is made available under aCC-BY 4.0 International license.

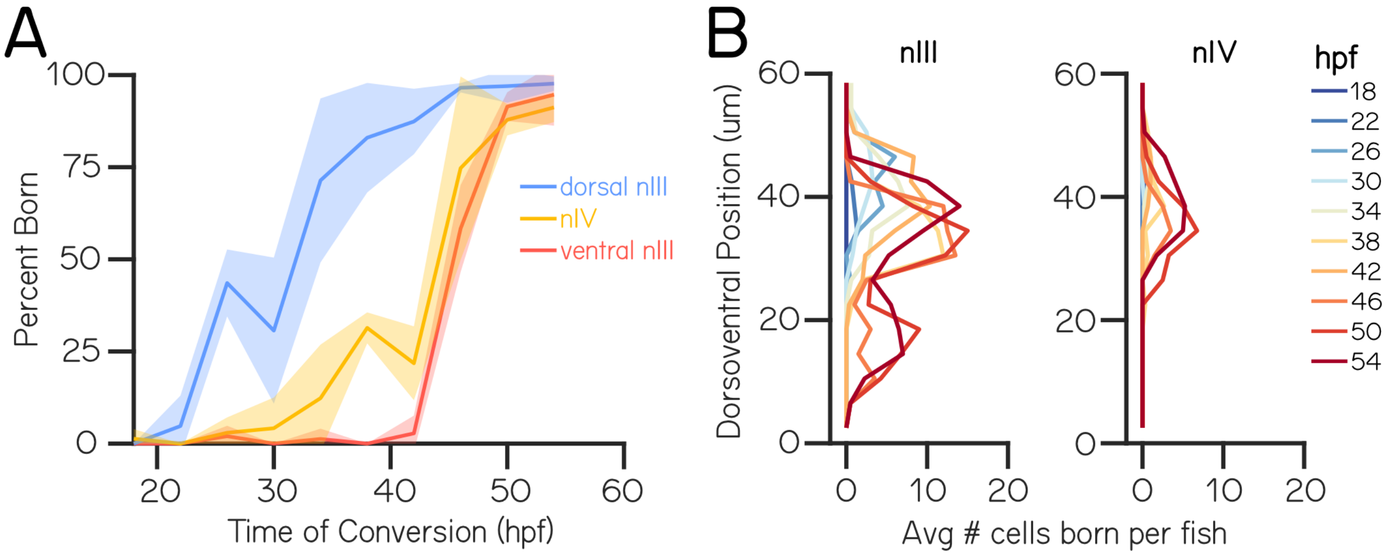

Figure S1: extraocular motor neuron birthdate varies with dorsoventral position

(A) Percent of *Tg(isl1:Kaede)* neurons that were born prior to the time of photoconversion in dorsal nIII (blue), ventral nIII (red) and nIV (yellow). Lines are averages across all fish, ribbons represent ±1 standard deviation or range. N = 4 fish (18 hpf), 3 fish (22 hpf), 2 fish (26 hpf), 4 fish (30 hpf), 5 fish (34 hpf), 3 fish (42 hpf), 2 fish (46 hpf), 4 fish (50 hpf), and 4 fish (54 hpf)

(B) Dorsoventral position of photoconverted *Tg(isl1:Kaede)* neurons in nIII (left) and nIV (right) colored by photoconversion timepoint (18-54 hpf).

bioRxiv preprint doi: https://doi.org/10.64898/2026.04.07.717009; this version posted April 8, 2026. The copyright holder for this preprint (which was not certified by peer review) is the author/funder, who has granted bioRxiv a license to display the preprint in perpetuity. It is made available under aCC-BY 4.0 International license.

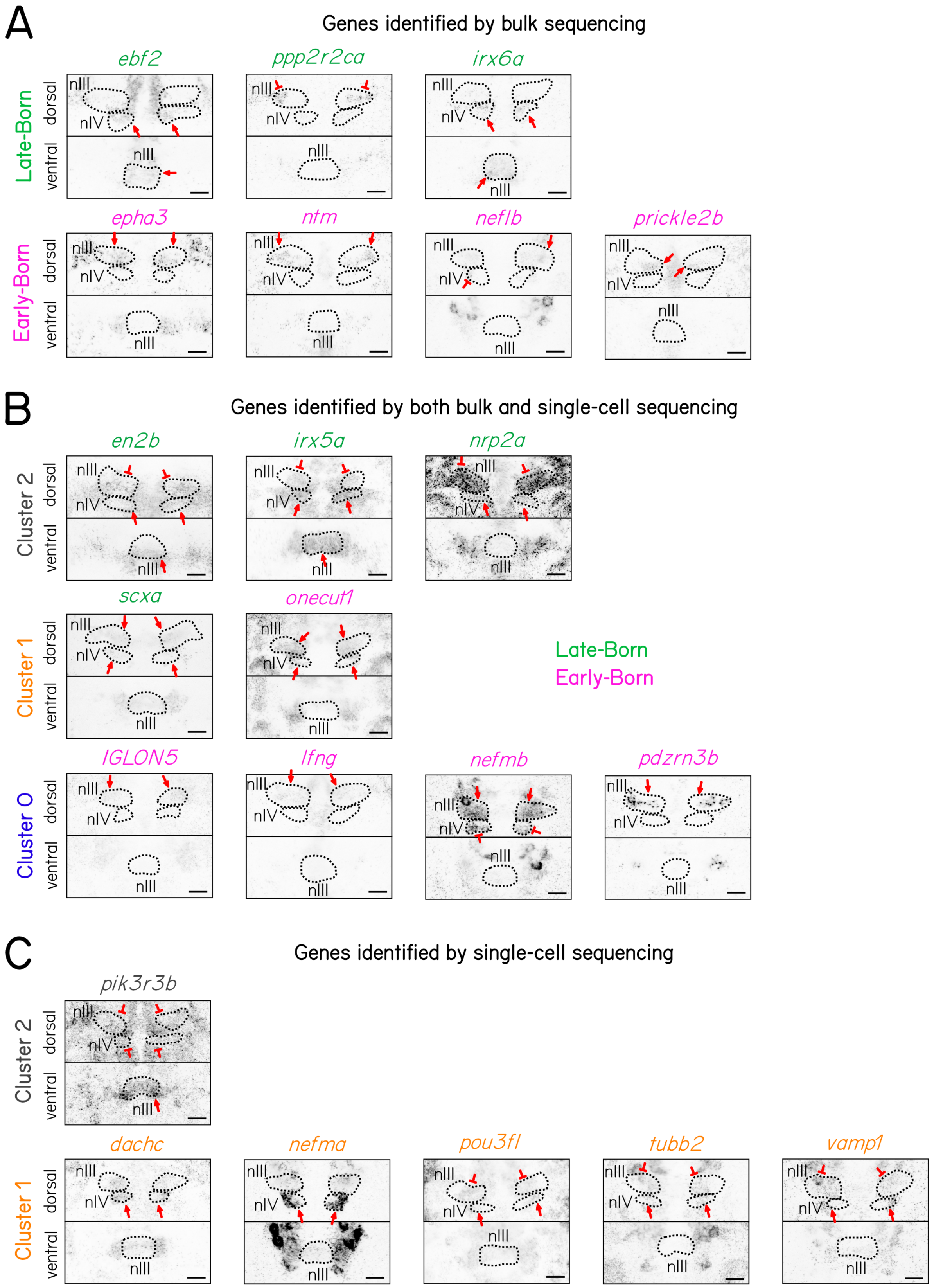

Figure S2: Fluorescent *in situ* hybridization expression for candidate genes identified in bulk and single-cell RNA sequencing of extraocular motor neurons in nIII and nIV. Maximum intensity projections of RNA probe expression in extraocular motor neurons split by dorsal (dorsal nIII and nIV) and ventral (ventral nIII) location. Black dashed outlines correspond to the location of extraocular motor neuron populations labeled by *Tg(isl1:Kaede)* (Kaede expression not shown). Scale bar 20 µm. **(A)** RNA probe expression for genes identified by bulk sequencing of late- (top, green) and early-born (bottom, pink) cells. Red arrows indicate expression in populations in which candidates were predicted to be enriched based on sequencing data. Red Ts indicate expression in other populations. **(B)** RNA probe expression for genes identified by both bulk and single-cell sequencing. Gene names are colored by late- (green) and early-born (pink) groups and organized by cluster (top: cluster 0, gray; center: cluster 1, orange; bottom: cluster 2, blue). Red arrows indicate expression in populations in which candidates were predicted to be enriched based on sequencing data. Red Ts indicate expression in other populations. **(C)** RNA probe expression for genes identified by single-cell sequencing. Genes are organized by cluster (top: cluster 0, gray; bottom: cluster 1, orange). Red arrows indicate expression in populations in which candidates were predicted to be enriched based on sequencing data. Red Ts indicate expression in other populations.

bioRxiv preprint doi: https://doi.org/10.64898/2026.04.07.717009; this version posted April 8, 2026. The copyright holder for this preprint (which was not certified by peer review) is the author/funder, who has granted bioRxiv a license to display the preprint in perpetuity. It is made available under aCC-BY 4.0 International license.

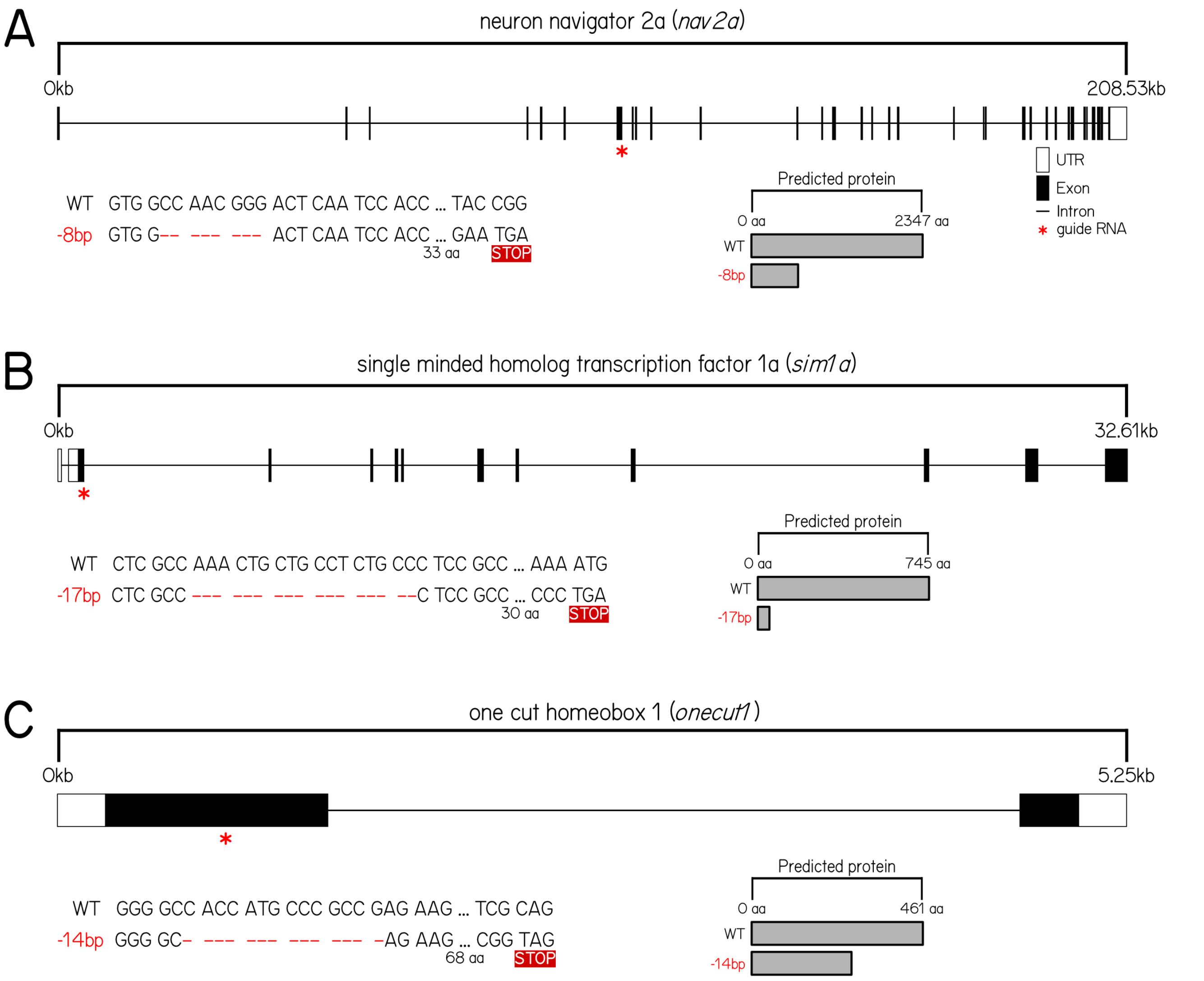

Figure S3: CRISPR/Cas9 mediated mutagenesis of select candidate genes
(A) Top: Schematic representing mutation created in *nav2a*. Red star indicates location of guides against nav2a DNA. Filled boxes show exons, open boxes show UTRs, and horizontal lines show introns. Bottom: Left shows DNA sequence in wildtype and *nav2a*^{d8} alleles. Red dashed lines indicate deleted sequence. STOP box indicates predicted premature stop codon due to deletion. Right shows predicted protein sequence.
(B) Top: Schematic representing mutation created in *sim1a*. Bottom: DNA sequence and predicted protein in wildtype and *sim1a*^{d17} alleles.
(C) Top: Schematic representing mutation created in *onecut1*. Bottom: DNA sequence and predicted protein in wildtype and *onecut1*^{d14} alleles.
